# Adenovirus vector delivery stimulates natural killer cell recognition

**DOI:** 10.1099/vir.0.82685-0

**Published:** 2007-04

**Authors:** Peter Tomasec, Eddie C. Y. Wang, Veronika Groh, Thomas Spies, Brian P. McSharry, Rebecca J. Aicheler, Richard J. Stanton, Gavin W. G. Wilkinson

**Affiliations:** 1 Department of Medical Microbiology, Cardiff University, Heath Park, Cardiff CF14 4XY, UK; 2 Department of Medical Biochemistry and Immunology, Cardiff University, Heath Park, Cardiff CF14 4XY, UK; 3 Fred Hutchinson Cancer Research Center, Clinical Research Division, 1100 Fairview Avenue North, Seattle, WA98109, USA

## Abstract

We report that delivery of first-generation replication-deficient adenovirus (RDAd) vectors into primary human fibroblasts is associated with the induction of natural killer (NK) cell-mediated cytolysis *in vitro*. RDAd vector delivery induced cytolysis by a range of NK cell populations including the NK cell clone NKL, primary polyclonal NK lines and a proportion of NK clones (36 %) in autologous HLA-matched assays. Adenovirus-induced cytolysis was inhibited by antibody blocking of the NK-activating receptor NKG2D, implicating this receptor in this function. NKG2D is ubiquitously expressed on NK cells and CD8^+^ T cells. Significantly, *γ*-irradiation of the vector eliminated the effect, suggesting that breakthrough expression from the vector induces at least some of the pro-inflammatory responses of unknown aetiology following the application of RDAd vectors during *in vivo* gene delivery.

Replication-deficient adenovirus (RDAd) vectors are used extensively as vehicles to promote *in vivo* gene expression both in experimental animal models and human gene therapy. However, the administration of RDAd vectors *in vivo* is associated with the induction of inflammatory responses (Schnell *et al.*, 2001), which have resulted in morbidity and mortality in gene therapy trials (Crystal *et al.*, 1994; Marshall, 1999; Raper *et al.*, 2003; Schnell *et al.*, 2001). Although the pro-inflammatory properties of adenovirus vectors are well documented, the molecular mechanisms responsible are not fully characterized (Liu & Muruve, 2003). Immediately following vector delivery, a transient early inflammatory response can be induced that is driven by innate mechanisms. This rapid host response effectively precedes *de novo* vector-encoded gene expression, and cannot be eliminated by irradiating RDAd vectors to render them transcriptionally inactive (Muruve, 2004). In contrast, delayed host responses tend to be mechanistically linked with expression from the vector, and may stimulate both the innate and adaptive immunity. Whether rapid or delayed, the pro-inflammatory effects tend to be dose-dependent and associated with the induction of a wide range of cytokines. Neutrophils, monocytes, macrophages, Kupffer and dendritic cells have all been implicated as important effector cells in various model systems (Muruve, 2004).

Whilst the underlying causes are complex, recent studies indicate that natural killer (NK) cells constitute a key component of the response to RDAd vectors *in vivo* (Marshall, 1999; Ruzek *et al.*, 2002; Xu *et al.*, 2004).

NK cells play a crucial role as a first line of defence against virus infection, responding to direct intercellular contact and the cytokine environment. They comprise a heterogeneous population, expressing a mosaic of inhibitory and activating cell-surface NK receptors. The integration of signals received through these NK receptors engaging their ligands on potential targets determines the response of any particular NK cell (Lanier, 2005). The characterization of NK receptors, in recent years, has provided a detailed insight into NK recognition. NK receptors belong to either the immunoglobulin superfamily (IgSF) or the C-type lectin-like superfamily (LSF). IgSF inhibitory receptors include the killer cell Ig-like receptors (KIRs) and the leukocyte Ig-like receptor-1 (LIR-1). KIRs recognize specific intact HLA-A, -B or -C complexes, whereas LIR-1 interacts broadly with all HLAs (Cosman *et al.*, 1997). LSF inhibitory receptors include CD94-NKG2A and CD161 (NKR-P1A), recognizing HLA-E and lectin-like transcript-1 (LLT-1), respectively (Aldemir *et al.*, 2005; Rosen *et al.*, 2005). The IgSF receptors also include a number of NK-activating members: CD226 (DNAM-1) recognizes CD112 and CD155, CD96 recognizes CD155, while CD224 (2B4) recognizes CD48 (Bottino *et al.*, 2003; Fuchs *et al.*, 2004; Nakajima *et al.*, 1999). The ligands for NKp30, NKp44, NKp46 and activating KIR isoforms have yet to be defined. The homodimeric activating receptor NKG2D recognizes at least seven distinct ligands (NKG2DL): the MHC class I-related chains MICA, MICB, UL16-binding proteins (ULBP) 1–3, ULBP4 (RAET1E) and RAET1G (Bacon *et al.*, 2004; Bauer *et al.*, 1999; Chalupny *et al.*, 2003; Cosman *et al.*, 2001). This property renders NKG2D particularly adept at identifying stressed cells, as its ligands can potentially be differentially upregulated by infection (viral or bacterial), DNA damage or heat shock (Bauer *et al.*, 1999; Gasser *et al.*, 2005; Groh *et al.*, 1996, 1998, 2001).

RDAd vectors are an invaluable laboratory tool for characterizing gene functions and we have exploited them extensively to analyse NK cell evasion functions (Fooks *et al.*, 1995; Griffin *et al.*, 2005; Tomasec *et al.*, 2000, 2005; Wills *et al.*, 2005). RDAd vector systems used to generate recombinant viruses for these studies were described elsewhere (He *et al.*, 1998; Wilkinson & Akrigg, 1992), AdEasy1 vector was kindly provided by Dr B. Vogelstein (Johns Hopkins University, USA) and pJM17 plasmid-based vector derived from human adenovirus type 5 (HAdV-5) *dl*309 was kindly provided by Dr F. Graham (McMaster University, Canada). Transgenes were inserted downstream of the human cytomegalovirus major immediate-early promoter (He *et al.*, 1998; McGrory *et al.*, 1988; Wilkinson & Akrigg, 1992).

Delivery of either AdEasy1 or pJM17-derived empty control vector could, in our previous studies, be sufficient by itself to stimulate killing of human fibroblasts by NK cells, independent of transgene expression. We set out to examine the effect of vector infection stimulating NK cytotoxicity in more detail. Human fetal foreskin fibroblasts (HFFF) were used for allogeneic NK cytotoxicity assays. Primary skin fibroblasts cultured from biopsies and immortalized using retroviral telomerase (hTERT) (McSharry *et al.*, 2001) were used for autologous NK cytotoxicity assays. NK clone NKL (kindly provided by Dr M. Robertson, Indiana University Medical Center, USA) was grown in RPMI, 1 % human heat-inactivated AB serum containing 200 U recombinant human interleukin-2 (IL-2) ml^−1^ (Robertson *et al.*, 1996). Primary polyclonal NK lines (designated D#NKp, ‘#’ indicates the donor number) were grown in RPMI supplemented with 5 % human heat-inactivated AB serum and 1000 U IL-2 ml^−1^, following depletion of T cells using the OKT3 monoclonal antibody and Dynabeads. They were regularly co-cultured with irradiated, allogeneic peripheral bloodmononuclear cells and immortalized B lymphoblastoid cell lines as feeders. NK polyclonal lines were >95 % CD3^−^CD56^+^ at use. NK cell cloning was carried out as previously described (Morris *et al.*, 2005). NK cytotoxicity was measured in standard 4 h ^51^Cr release assays; the final means and standard deviations were determined from triplicate or quadruplicate samples.

RDAd vector delivery to human fibroblast targets reproducibly stimulated cytolysis by polyclonal NK lines from all donors tested (Fig. 1a[Fig f1]). To further investigate the breadth of the vector effect, we analysed 81 NK clones from three donors. To be included in analysis, NK clones had to exhibit cytotoxicity against K562 targets (>10 % ^51^Cr release). Then, to be scored as responding to RDAd infection, the NK clone killing had to be either induced or inhibited by >10 % compared to mock infection (Wills *et al.*, 1996). The RDAd vector effect on individual NK cell clones was more variable than on polyclonal lines, yet activation by RDAd or RDAd–GFP vector delivery was consistently observed from a large proportion of clones. Data from a single donor (D8) are shown in Table 1 and indicate the reproducibility of the effect from clones tested multiple times. Combining the results from all three donors, 36 % (29 of 81) of NK clones were stimulated upon encounter with autologous RDAd-infected targets, while none were inhibited (Table 2). The frequency of clones stimulated to kill RDAd-infected targets revealed that a substantial proportion of NK cells were sensitive to this effector mechanism.

The binding of adenovirus to its cellular receptors and subsequent uptake by receptor-mediated endocytosis is known to stimulate intracellular signalling events (Borgland *et al.*, 2000; Li *et al.*, 1998; Rafii *et al.*, 2001). Specifically, adenovirus binding with its secondary integrin receptor during endocytosis is known to induce a transient activation of NF-*κ*B (Borgland *et al.*, 2000; Philpott *et al.*, 2004). This response was detected in RDAd-infected fibroblasts, but was short lived and preceded the time points when NK assays were performed. *In vivo* pro-inflammatory effects have also been attributed to breakthrough expression from first-generation RDAd vectors (Muruve *et al.*, 2004). GFP-encoding RDAd was *γ*-irradiated with the minimal dosage required to eliminate transgene expression, yet still permitted binding and uptake of virus particles into fibroblasts. The ability of the *γ*-irradiated virus to retain binding to HFFF was demonstrated by comparison of HFFF infected with normal or *γ*-irradiated viruses labelled with fluorescent DNA dye YOYO-1 (not shown). The *γ*-irradiated virus lost the capacity to stimulate NKL killing (Fig. 1b[Fig f1]). Priming of NK recognition was therefore generated by a post-entry event requiring an intact RDAd vector genome, thus implicating breakthrough expression from the RDAd vector. Furthermore, conditioned media from RDAd-infected fibroblasts had no obvious effect in these assays (Fig. 1c[Fig f1]), indicating that vector-induced NK cell killing was not driven by secreted factors. To investigate the mechanism responsible for vector-induced NK cell recognition, assays were performed in which characterized NK cell receptor interactions were blocked with specific antibodies. Indeed, blocking of the NKG2D interactions with its ligands using anti-NKG2D antibody 1D11 (Groh *et al.*, 1998) could reverse the stimulating effect of RDAd infection upon NKLs (Fig. 1d[Fig f1]) or a number of polyclonal NK lines derived from different donors (Fig. 1e[Fig f1]). Thus, the engagement of NK-activating receptor NKG2D with its ligands appeared to be a factor in promoting NK cell lysis of RDAd-infected cells. The NK-activating receptor NKG2D recognizes at least seven ligands (as described above), each capable of independently activating the NKG2D receptor. When the 6D4 (Groh *et al.*, 1998) antibody to MICA/B was used, it could only partially reverse the RDAd vector-induced activation of NK killing (not shown), suggesting that RDAd infection upregulated multiple NKG2D ligands. Indeed, semiquantitative RT-PCR analysis of RDAd-infected cells indicated that the levels of MICA/B and ULBP1-3 were all enhanced following RDAd infection of human fibroblasts (Fig. 1f[Fig f1]).

This study was instigated in response to an initial observation that RDAd vector delivery was routinely associated with enhanced NK cell-mediated killing in *in vitro* cytotoxicity assays. The effect was eliminated by irradiation of the RDAd vector. NK cell killing was stimulated irrespective of the transgene encoded by the RDAd vector and in the absence of transgene, thus indicating that it was induced by breakthrough expression from the RDAd vector ‘backbone’. The effect could readily be detected in either an autologous or an allogeneic setting and using a diverse range of NK cells as effectors, including NKL cells, expanded NK lines and a substantial proportion of NK clones tested. The underlying mechanism therefore clearly affects a high proportion of the effector NK cells. Although NK cells are a heterogeneous population of cells expressing a wide variety of activating and inhibitory receptors, NKG2D is expressed ubiquitously. The level of NKG2DL expression induced by RDAd vectors was sufficient to induce a clear dominant activating signal triggering cytolysis in 36 % of NK clones tested. The strict requirement for a change of 10 % in the absolute level of cytolysis for a clone to be scored may well underestimate the level of activation. Nevertheless, for a substantial proportion of clones, the upregulation on NKG2DL was not sufficient to override other inhibitory signals received from the targets.

The *in vivo* pro-inflammatory properties of adenovirus vectors can rationally be differentiated into ‘rapid’ effects, mediated by direct interactions of virus particles, and ‘delayed’ effects, associated with *de novo* expression from the vector and/or its transgene. The enhancement of NK killing of RDAd-infected targets is clearly mediated by breakthrough expression from the vector, thus its elimination could be expected to only diminish the delayed response. Avoiding vector-associated inflammation is highly desirable for somatic monogenic replacement therapies; however, the vast majority of current adenovirus vector applications are concerned with anti-tumour therapies or immunization protocols. In such circumstances, the induction of NK lysis could be postulated to be therapeutically beneficial in stimulating direct killing or specific immunity to endogenous tumour- and vector-expressed antigens. In this context, the delivery of RDAd vectors lacking an insert or encoding NKG2D ligands have already been shown to promote tumour cell rejection in murine models (Friese *et al.*, 2003; Ruzek *et al.*, 2002). Manipulation of replication-deficient, replication-competent and/or oncolytic vector systems so as to accentuate this natural property of the virus to activate NKG2D could potentially enhance their therapeutic efficacy in certain circumstances.

Productive virus infections have been reported to activate the expression of NKG2D ligands; in this context, both human and murine CMV encode NK evasion functions that act specifically to combat their induction (Cosman *et al.*, 2001; Dunn *et al.*, 2003; Lanier, 2005; Welte *et al.*, 2003). However, RDAd vectors are by definition ‘replication-deficient’ and their delivery to primary human fibroblasts is relatively benign, in that they induce neither ‘substantial’ vector-specific gene expression nor a cytopathic effect (Wilkinson & Akrigg, 1992). In this study, the RDAd vector did not induce a heat-shock response (not shown) and was associated with only limited perturbation of NF-*κ*B signalling. The existence of multiple NKG2D ligands is consistent with a single activating receptor being sensitive to multiple stimuli. The potential for coordinated upregulation of multiple NKG2D ligands by vector delivery was therefore surprising, and suggests that this response could have evolved to be exquisitely sensitive to detecting the early phase of virus infection. In this context, it is important to note that HAdV-5 E1A gene has recently been shown to upregulate NKG2D ligands via interaction with p300 (Routes *et al.*, 2005). Since E1A is deleted from all RDAd vectors used in this study, HAdV-5 must encode more than one function that activates the expression of NKG2D ligands.

Antibody-blocking experiments were consistent with the activation of NKG2D being the primary mechanism responsible for RDAd-induced NK cell-mediated cytolysis. Nevertheless, it remains feasible that the RDAd vector may yet prove capable of stimulating NK recognition by other means. These issues are currently being addressed. This study has been concerned with RDAd vector induction of NK cell recognition; however, it should not be ignored that human NKG2D is also ubiquitously expressed on T cells and interferon-producing killer dendritic cells. NKG2D ligands can substitute for CD28 to act as a co-stimulatory molecule to promote CD8^+^ CTL effector cell functions and can act in isolation to stimulate *γδ* T cell function. The activation of NKG2D by RDAd vectors could therefore be expected to influence T cell responses directly and also have a profound effect on stimulating both innate and adaptive immune responses through the release of cytokines.

## Figures and Tables

**Fig. 1. f1:**
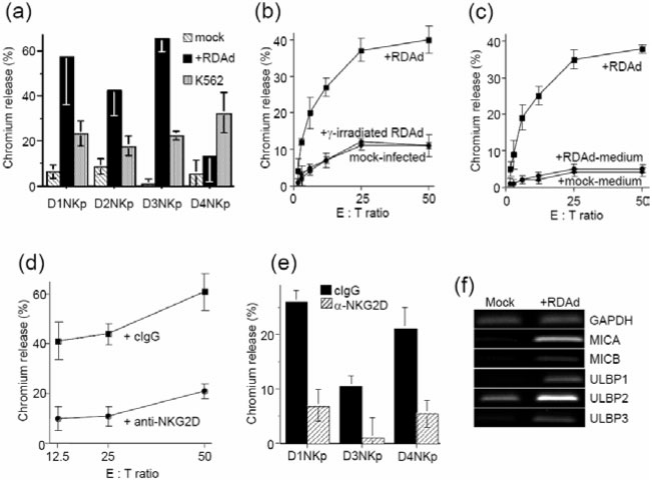
Induction of NK cell-mediated cytolysis following RDAd infection. (a) Susceptibility of human fibroblasts infected for 72 h with a control vector with no transgene insertion (+RDAd) to cell-mediated cytolysis of polyclonal NK lines; autologous assays, K562 cells and mock-infected fibroblasts were used as cytotoxicity points of reference. (b) Infection of HFFF with RDAd and not with *γ*-irradiated RDAd induced NKL killing [72 h post-infection (p.i.)]. E : T Ratio, effector : target cell ratio. (c) Susceptibility to NKL cells was not increased by soluble factors (RDAd-medium) secreted from infected cells (72 h p.i.). (d) NKG2D interactions and NKL killing were blocked efficiently with the anti-NKG2D antibody. (e) Similar results were observed using polyclonal NK lines in autologous assays. (f) mRNA levels of multiple NKG2D ligands were enhanced following RDAd infection using semiquantitative RT-PCR.

**Table 1. t1:** Susceptibility of RDAd-infected cells to NK cell-mediated lysis Autologous assays in which values are given as percentage of ^51^Cr released±sd. E : T Ratio, effector : target cell ratio.

**NK clone**	**E : T Ratio**	**Mock**	**+RDAd**	**+RDAd–GFP**	**K562**
D8.NKc1C10*	58 : 1	48±2	53±3	−	63±1
	8 : 1	13±2	21±1	−	32±3
	27 : 1	22±2	−	39±1†	56±4
D8.NKc1E11*	21 : 1	29±2	34±1	−	48±4
	40 : 1	10±1	−	33±4†	52±3
	23 : 1	24±1	18±1	−	46±5
D8.NKc1F11*	44 : 1	24±2	38±2†	−	52±3
	32 : 1	7±1	19±1†	−	31±1
	19 : 1	12±1	−	23±1†	35±1
D8.NKc2F3*	25 : 1	38±3	45±2	−	49±5
	20 : 1	22±1	−	31±3	37±2
D8.NKc2G9	5 : 1	2±1	3±1	−	23±2
D8.NKc3C8*	26 : 1	8±1	12±4	−	29±3
	35 : 1	4±2	24±1†	−	40±4
	20 : 1	11±2	−	21±1†	46±3
D8.NKc3D9	11 : 1	11±1	19±3	−	34±3
D8.NKc3E9*	32 : 1	35±1	31±1	−	28±4
	18 : 1	7±1	−	11±1	30±3
D8.NKc4F3*	25 : 1	48±2	40±6	−	52±1
	10 : 1	24±1	−	39±1†	61±4
D8.NKc4E10*	44 : 1	13±3	26±3†	−	40±4
	23 : 1	11±2	30±3†	−	41±4
	20 : 1	6±1	−	16±2†	30±3
D8.NKc5E7	14 : 1	7±3	5±3	−	19±2
D8.NKc5C11*	63 : 1	36±2	43±3	−	55±2
	66 : 1	19±2	36±3†	−	51±3
	50 : 1	24±3	−	34±3†	59±4

*Multiple assays carried out at different times showing stability of clonal response. †Increase in killing of RDAd-infected cells was greater than 10 %.

**Table 2. t2:** Effect of RDAd infection on cytotoxicity mediated by individual NK clones derived from multiple donors Autologous assays; each event represents results obtained from an individual clone (clones tested more than once were averaged as a single event, some clones were tested against RDAd–GFP, as in Table 1).

**Donor**	RDAd infection increased lysis*	RDAd infection inhibited lysis*	**RDAd infection had no effect†**
CD94^hi^ clones			
D3	3/4	0/4	1/4
D8‡	4/13	0/13	9/13
D9	22/64	0/64	42/64
Total	29/81 (36 %)	0/81	52/81 (64 %)

*Increase or decrease in cytolysis was greater than 10 %. †Increase or decrease in cytolysis was less than 10 %. ‡Complete results using this donor are presented in Table 1.
